# Research Progress and Trends in Remote-Sensing Retrieval of Water-Quality Parameters: A Knowledge Graph Analysis

**DOI:** 10.3390/s26082335

**Published:** 2026-04-09

**Authors:** Hongbo Li, Xiuxiu Chen, Shixuan Liu, Conghui Tao, Qiuxiao Chen

**Affiliations:** 1 School of Spatial Planning and Design, Hangzhou City University, Hangzhou 310015, China; lhb@hzcu.edu.cn (H.L.);; 2 Laboratory for Microwave Spatial Intelligence and Cloud Platform, Hangzhou 310015, China; 3 Eco-Environmental Science Research & Design Institute of Zhejiang Province, Hangzhou 310007, China; sxliu1994@163.com; 4 Water Pollution Control Engineering Technology (Zhejiang) Center, Ministry of Ecology and Environment, Hangzhou 310007, China; 5 Siwei Gaojing Satellite Remote Sensing Co., Ltd., Hangzhou 310012, China; taoconghui@swgjsatellite.com

**Keywords:** water-quality parameters, remote-sensing inversion, CiteSpace, narrative review, knowledge graph analysis

## Abstract

Remote-sensing inversion of water-quality parameters is a critical interdisciplinary field, integrating remote-sensing technology, environmental science, and water resources management, providing key technical support for precise water resources monitoring and ecological governance. To address the lack of comprehensive systematic reviews in this field, this study conducted a bibliometric-based narrative review, selecting 2812 valid English studies published during 1980–2026 from the Web of Science Core Collection (WOSCC) and performing in-depth knowledge mapping analysis via CiteSpace software. The results showed that global research in this field has gone through three stages: initial exploration (1980–2000), slow growth (2001–2015), and rapid explosion (2016–2026). China ranks first in publication volume worldwide, with a collaborative research pattern dominated by core institutions, including the Chinese Academy of Sciences, Wuhan University, and the National Aeronautics and Space Administration (NASA). The core research hotspots focus on multi-source data fusion, AI-driven inversion-model optimization, and the research shift from coastal to inland water bodies. Current research faces three key challenges: poor adaptability of multi-source data-fusion technologies to water-quality monitoring, inadequate integration of geospatial and thematic factors in inversion models, and an insufficient systematic approach of inland-water-body research. Accordingly, future research should focus on advancing remote-sensing data-fusion methods, further optimizing water-quality inversion models, and strengthening inland-water-body studies. This study clarifies the field’s development context and research characteristics, providing valuable references for subsequent academic exploration and practical applications in water resources management.

## 1. Introduction

As strategic resources for maintaining ecosystem balance and supporting the sustainable development of the economy and society, water resources have always been a core component of the national water security guarantee system. Ensuring water quality and safety, as well as the rational utilization of water resources, has become a global consensus. In 1993, the United Nations established the “World Water Day” to address the global water resources crisis, adopted resolutions to promote the protection and scientific management of water resources, and raise public awareness of water resource issues. Since 1994, China has designated 22 to 28 March as “China Water Week”, and has attached great importance to water resources management. Top-tier documents, such as the State Council’s Regulations on Water Conservation and the National Development and the Reform Commission’s Opinions on Further Strengthening Water Resources Management, have clearly proposed the “Four Waters and Four Determinations” principle (“Four Waters and Four Determinations” is the abbreviation for “determining urban development, land use, population size, and industrial production in accordance with water availability”. This principle emphasizes treating water resources as the most rigid constraint, aiming to achieve intensive water resource utilization by optimizing territorial spatial development patterns, controlling population and industrial scales, and promoting water-saving technologies) and the “Three Red Lines” institutional framework (“Three Red Lines” refers to the three control lines for water resources management: control over water resource development and utilization, control over water-use efficiency, and restriction on pollutant discharge into water functional zones), elevating the protection and rational utilization of water resources to the national strategic level. Against this backdrop, the demand for precision and scientificity in water resources management has become increasingly urgent. As a core foundational link for grasping water body conditions and supporting water resources management, the technical upgrading of water-quality monitoring has emerged as a key breakthrough point.

Currently, in response to the demand for precise and scientific water resources management, water resources research is moving towards an integrated direction of “refined dynamic monitoring—multi-factor coupling analysis—risk early warning and forecasting” [1,2,3]. Among these, the water resources monitoring system serves as the core support for water resources management, with its key breakthrough lying in refined, dynamic water-quality-monitoring technologies. However, traditional monitoring methods are increasingly unable to meet the aforementioned monitoring requirements, manifested in two aspects: firstly, insufficient spatiotemporal coverage capability, as single-point sampling fails to effectively capture large-scale water-quality-change processes; secondly, imbalanced cost-effectiveness, as field sampling requires substantial human and material resources for cross-regional and large-scale water-quality monitoring [4,5,6,7,8]. Therefore, the bottleneck of traditional monitoring methods has become prominent, and there is an urgent need for innovative technical means to promote the upgrading of the water-quality-monitoring system to adapt to the high standards of water resources management.

The emergence of remote-sensing technology has provided a new path for breaking through the bottlenecks in water-quality monitoring. Relying on the unique advantage of “air–space–ground coordinated observation,” large-scale, continuous, and rapid monitoring of water bodies can be conducted to collect abundant spectral information. This has led to the development of an efficient monitoring technology known as “remote-sensing-based water-quality monitoring,” offering significant breakthroughs and opportunities for advancing large-scale and precise water-quality management [9,10]. As an important technical extension of water-quality monitoring, remote-sensing-based water-quality monitoring refers to the technical process of obtaining water spectral information through remote-sensing technology to retrieve water-quality parameters and monitor water-quality conditions. It specifically includes links such as data collection (remote-sensing data and ground-measured data), data preprocessing, feature extraction, inversion model construction and parameter retrieval, accuracy verification, and result analysis and application [11,12]. With the development of remote-sensing technology and the improvement in hyperspectral resolution, the types of data sources for remote-sensing-based water-quality monitoring have become increasingly abundant, and their application potential has been continuously realized. In 1974, Strong [13] published his research paper on remote sensing of water quality, in which he first observed algal blooms in lakes using the ERTS-1 satellite. This paper laid an important foundation for the development of remote-sensing technology in the field of water-quality retrieval, and is widely recognized as one of the early pioneering studies in the field of water-quality remote sensing. Carpenter [14] successfully monitored water-quality parameters of an entire lake by analyzing the correlation between measured water-quality parameters from 21 sampling points and the spectral features of Landsat Multispectral Scanner remote-sensing images. With the development of remote-sensing technology and hyperspectral refinement, the types of remote-sensing data sources have been increasing. Yao [15] utilized the domestically developed Ziyuan-1 02E satellite, equipped with a new-generation hyperspectral camera (30 m spatial resolution/visible-shortwave infrared/166 bands), to successfully complete quantitative research on chlorophyll-a concentration and transparency in Shahe Reservoir and Jinhai Lake in Beijing, China, fully demonstrating the important potential of domestic hyperspectral data sources in water-quality remote-sensing monitoring.

Throughout the entire process of remote-sensing-based water-quality monitoring, water-quality remote-sensing inversion serves as the core technical step. It directly determines the accuracy and reliability of water-quality-parameter acquisition and provides key technical support for refined water resources management. Its principle is to establish a water-quality inversion model by studying the relationship between measured water-quality-index concentrations and scattering signals (wavebands) from satellite/Unmanned Aerial Vehicle (UAV) sensors, using large amounts of actual data to estimate model parameters to improve retrieval accuracy, and then inferring the spatiotemporal distribution of water-quality parameter concentrations across the entire study area [16,17,18]. Therefore, this paper will focus on remote-sensing retrieval of water-quality parameters and conduct an in-depth study of the literature in this direction.

In recent years, the knowledge-graph analysis method based on CiteSpace has become increasingly prominent and widely applied in the field of scientific research [19,20,21,22]. With its powerful data-processing capabilities, CiteSpace can perform in-depth mining and detailed analysis of the massive literature data, clearly present the knowledge structure of a specific research field, accurately depict the development context of the field, and vividly show the evolution of research hotspots over time. Thus, it provides researchers with a comprehensive and intuitive research perspective, helping them accurately grasp research trends.

Currently, research on the remote-sensing inversion of water-quality parameters is booming rapidly, yet a comprehensive and systematic review of this field is still lacking. Based on CiteSpace 6.4 software (hereinafter referred to as CiteSpace), this study conducts an in-depth and systematic analysis of the latest research advances in the field of remote-sensing inversion of water-quality parameters. From the perspectives of annual publication volume, national and institutional distribution, journal and reference co-citation, and keyword evolution, we systematically sort out the major research achievements, core research hotspots, evolutionary characteristics of research focus, and overall development trends of this field, focusing on analyzing in-depth the evolutionary trajectory of multi-source data-fusion technology, the iterative progression of remote-sensing-inversion methods, as well as the variation characteristics of typical research areas, and further put forward targeted prospects for future research in this field. Through the systematic bibliometric analysis of the relevant literature, this study accurately captures the cutting-edge research dynamics of the remote-sensing inversion of water-quality parameters, clarifies the core challenges faced by current research and the mainstream development trends in the future, and thus provides valuable references for subsequent studies on dynamic water-quality monitoring.

## 2. Materials and Methods

### 2.1. Data Sources

The Web of Science Core Collection (WOSCC), developed by Clarivate Analytics, is a world-class academic-citation database. Due to its comprehensive coverage of high-quality scholarly publications across various disciplines and reliable data integrity, it is widely recognized as one of the preferred data sources for bibliometric analysis [23]. In view of this, this study systematically analyzes the research progress in the field of remote-sensing retrieval of water-quality parameters based on the literature retrieved from WOSCC.

The quality of the literature retrieval largely affects the validity and accuracy of bibliometric analysis [24]. Focusing on the theme of remote-sensing retrieval of water-quality parameters, this study repeatedly calibrated the search terms and compared the retrieved results multiple times to verify their relevance to the research topic, thereby determining the final literature search strategy and content.

A topic-based search method was adopted, with the search formula set as TS = (“Water quality”) AND TS = (inversion OR retrieval OR model OR regression OR fitting) AND TS = (remote sensing OR Landsat OR Sentinel OR UAV OR Drone OR Multispectral OR Hyperspectral OR Virrs OR SeaWiFS OR Pace OR HY OR Envisat OR ERTS), with the document types limited to research articles and reviews. This search retrieved a total of 3566 documents, with publication dates spanning from 1980 to 2026. After excluding duplicate studies and those irrelevant to the research topic, a total of 2812 valid English studies were finally obtained.

### 2.2. Methods

This study employs CiteSpace 6.4 as the research tool, integrating bibliometric analysis and knowledge graph methodology. To ensure the reproducibility of the research, key parameter configurations were clearly specified as follows: the time slice size was set to 1 year to capture annual research trends with high granularity, the node selection parameter was set to k = 25 to ensure the relevance and representativeness of the included nodes, and the Pruning sliced-networks strategy was adopted to simplify the visualization network while preserving core structural information. Based on the above configurations, this study focuses on the scholarly publication output, national and institutional distribution, academic networks, and thematic evolution in the field of remote-sensing retrieval of water-quality parameters. It systematically analyzes the knowledge structure, hot topic dynamics, and development trends of the field through multi-dimensional quantitative and visual analysis, providing references for academic exploration and practical applications. The specific methods are as follows:

(1) National Analysis: It is a quantitative research method focusing on the global geographical distribution of research and international cooperation patterns. Constructing a cooperation network with countries/regions of authors as units (node size corresponds to publication volume, and line thickness corresponds to cooperation intensity), it can present the “core–periphery” structure of global research, reveal international cooperation models and network structures, and identify research expertise and characteristic directions of different regions through dimensions such as publication volume ranking, cooperation intensity, betweenness centrality, and regional-research thematic differences.

(2) Institutional Analysis: It is a quantitative method for evaluating the research output, cooperation networks, and academic influence of scientific research institutions. After standardizing institutional names, a cooperation network is constructed (node size corresponds to publication volume, and line thickness corresponds to collaborative publication volume). Through dimensions including publication volume, citation frequency, cooperative-network characteristics, and institution–thematic correlation, it can sort out the distribution of core research institutions in the field, reveal the collaborative ecology among institutions, clarify the research expertise of core institutions, and provide a basis for research cooperation docking.

(3) Co-Cited Journal Analysis: It is a method to quantify the co-citation relationships between journals and analyze their academic influence and disciplinary correlation. Constructing a network based on the co-citation data of source journals, combined with dimensions such as citation frequency, betweenness centrality, disciplinary attributes, and network density, it can identify the core journal cluster in the field, reveal interdisciplinary integration trends, provide references for researchers in selecting submission journals, and identify the core carriers of knowledge dissemination in the field.

(4) A Co-Cited Literature Analysis: It is a method to analyze the academic inheritance and correlation intensity between studies based on the co-citation relationship, where “two studies are simultaneously cited by the same subsequent literature”. Constructing a co-citation network using reference lists as data sources (node size corresponds to citation frequency, and line thickness corresponds to co-citation intensity), it can identify foundational achievements and key technological breakthrough nodes in the field, sort out the evolutionary path of academic thoughts, and clarify the formation and transformation of research paradigms through dimensions such as citation frequency, betweenness centrality, and citation temporal characteristics.

(5) Keyword Co-Occurrence Analysis: It is a method of constructing networks and conducting quantitative analysis based on the co-occurrence relationships of keywords in the literature. As a concise expression of the core content of the literature, keyword co-occurrence frequency and intensity directly reflect the correlation degree of research topics [25]. By constructing a network through statistical co-occurrence frequency (node size corresponds to keyword frequency, and line thickness corresponds to co-occurrence intensity), and combining dimensions such as frequency, betweenness centrality, and time-series evolution, it can effectively identify core hot topics in the field, reveal the correlation structure between themes, sort out the overall research pattern, and trace the evolution trajectory of hot topics [26].

(6) Keyword Burst Analysis: It is a frontier identification method that focuses on burst keywords with sharply increasing or decreasing frequencies within a specific time window to capture emerging trends in the field. Using the burst detection algorithm to calculate burst strength for quantifying the magnitude of frequency mutations, and dividing the life cycle of hot topics based on the start and end times of bursts, it can quickly lock in emerging research hotspots, predict potential research directions, and reveal the driving factors behind the rise in frontier issues [27].

(7) Keyword Clustering Analysis: It is a method to visually decompose the field’s knowledge structure by aggregating keywords with closely related semantics and similar research themes into several clusters using clustering algorithms such as Latent Semantic Indexing (LSI), Log-Likelihood Ratio (LLR), and Latent Semantic Analysis (LSA). The validity of clustering results is verified by two core indicators: ① Modularity Q (Q > 0.5 indicates significant cluster structure); ② Weighted Mean Silhouette S (S > 0.7 indicates reliable and efficient clustering). Combined with cluster size and labels, it can systematically sort out the knowledge branch system of the field, clarify the research focus and boundaries of each sub-field, and intuitively present the integrity and correlation of the knowledge structure [28].

## 3. Results

### 3.1. Global Publication Trend

**Figure 1:**
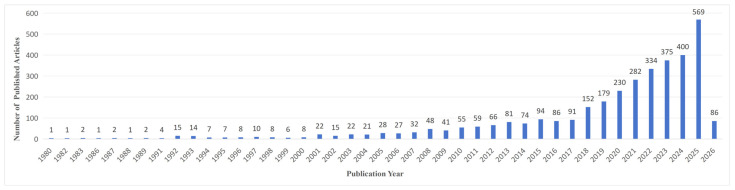
Publication trend of research on remote-sensing inversion of water-quality parameters.

The time span of the literature selected in this study was set from 1980 to 2026. The statistical results of annual publication volumes are shown in Figure 1. The trend of annual publication volumes in the field of remote-sensing retrieval of water-quality parameters can be clearly divided into three stages: the initial exploration stage (1980–2000), the slow growth stage (2001–2015), and the rapid explosion stage (2016–2026).

During the initial exploration stage, this field was still in the early embryonic period. The core technical system, including remote-sensing retrieval algorithms and water-quality-monitoring data sources, had not yet taken shape. Academic attention and research investment remained limited, and the scale of publication volumes stayed at a low level for a long time. However, it is noteworthy that there was a minor peak in the number of publications in this field in 1992 and 1993.

In the slow growth stage, the annual publication output remained around 20 papers in the first five years. During this period, the gradual popularization of remote-sensing technology and the continuous increase in demand for water-quality environmental monitoring drove a steady rise in research attention to this field, thus facilitating the initial expansion of relevant studies. However, constrained by technical maturity and application scenarios, the overall growth momentum remained relatively moderate.

The publication output during the rapid explosion stage showed a trend of explosive growth, with annual publication volumes exceeding 100 since 2018 and reaching 569 in 2025, which was mainly driven by multiple key factors. First, breakthroughs in core technologies, such as the introduction of machine learning (including artificial intelligence algorithms) and the popularization of hyperspectral remote-sensing and UAV monitoring data, provided strong support for the innovative development of the field. Second, the growing global attention to water-quality environmental governance formed a robust policy-driven impetus. In addition, the increasingly prominent trend of interdisciplinary integration among remote-sensing science, environmental science, and computer science further boosted the upsurge of research. The growth momentum during this stage indicates that the field of remote-sensing retrieval of water-quality parameters has entered a period of high academic activity.

### 3.2. Analysis of Country and Region Distribution of Published Articles

**Figure 2:**
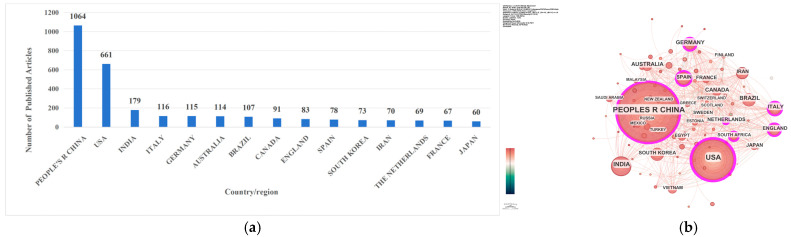
Analysis of the country/region distribution of global publications in the field of remote-sensing inversion of water-quality parameters: (**a**) top 15 countries/regions by publication volume; (**b**) network visualization map.

From the publication volume distribution in Figure 2a, research in this field has spread across multiple countries, with variations in the level of participation among different nations, which demonstrates strong global research coverage. China ranks first with 1064 publications, followed by the United States with 661 publications, and India comes in third with 179 publications. Additionally, four other countries, Italy, Germany, Australia, and Brazil, have each published over 100 articles.

Regarding the country collaboration network map in Figure 2b, it consists of 114 nodes and 782 connections, with a network density of 0.1214, a modularity Q of 0.4836, and a weighted mean silhouette S of 0.7981. These metrics reflect that certain collaborative links have been established among countries in this field, presenting an international research interaction pattern involving diverse participants.

### 3.3. Analysis of Institutions’ Distribution of Published Articles

**Figure 3:**
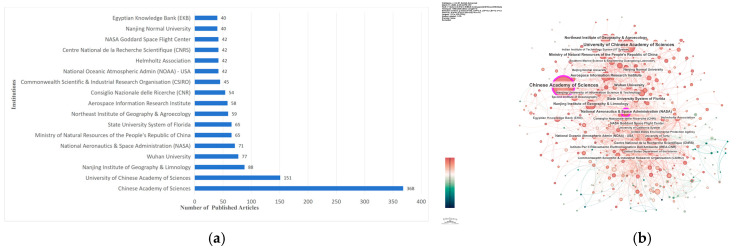
Analysis of the institutional distribution of global publications in the field of remote-sensing inversion of water-quality parameters: (**a**) top 17 institutions by publication volume; (**b**) network visualization map.

Based on data from the WOSCC database, 619 institutions (over 600) have published relevant research papers in the field of remote-sensing inversion of water-quality parameters. Figure 3a focuses on institutions with more than 40 publications. Among them, the Chinese Academy of Sciences, University of the Chinese Academy of Sciences, Nanjing Institute of Geography & Limnology, Wuhan University, the National Aeronautics and Space Administration (NASA), the State University System of Florida, Consiglio Nazionale delle Ricerche (CNR), the Commonwealth Scientific & Industrial Research Organisation (CSIRO), and the Centre National de la Recherche Scientifique (CNRS) are all top publishing institutions in this field.

The institutional collaboration map corresponding to Figure 3b consists of 619 nodes and 2131 connections, with a network density of 0.0111. This map visually depicts the collaboration network among institutions: the denser or thicker the connections between nodes, the stronger the collaborative ties between the corresponding institutions. This feature also reflects that research in the field of remote-sensing inversion of water-quality parameters not only relies on the leading role of core institutions, but also forms an interactive pattern of multi-institutional collaborative participation.

### 3.4. Analysis of Co-Cited Journals

A co-cited journal map is a valuable tool for researchers, enabling them to evaluate the influence and status of different journals [29]. As shown in Table 1, in terms of the number of publications, the academic journals *Remote Sensing of Environment*, *International Journal of Remote Sensing*, and *Remote Sensing* have relatively high publication volumes, with 2267, 1841, and 1775 articles, respectively. It is demonstrated that these three international journals enjoy a high standing in the field of remote-sensing inversion of water-quality parameters. In addition, the journals *Science of the Total Environment*, *Limnology and Oceanography*, *IEEE Transactions on Geoscience and Remote Sensing*, and *Water* (Switzerland) have each published over 1000 articles in this field.

### 3.5. Analysis of the Co-Cited Literature Network

Within the framework of the co-citation literature network analysis, the literature continuously cited at a high frequency is usually defined as the classic literature in this field. This characteristic can not only clearly reflect the development context of the field of remote-sensing inversion of water-quality parameters, but also reveal the core research foundation of this field. Table 2 lists the top 10 research papers ranked by co-citation frequency, and also indicates the number of citations of each paper in the WOS database. From the perspective of the title characteristics of these 10 papers, studies focusing on chlorophyll inversion account for a relatively prominent proportion; in addition, papers conducting analysis based on Sentinel-2 satellite data also make up a considerable proportion. Based on the analysis of the top 10 highly cited publications, the field exhibits the following characteristics: In terms of temporal distribution, research hotspots are concentrated in the period of 2020–2022, while earlier seminal studies (such as those published in 2016) continue to exert sustained influence. Thematically and methodologically, the integration of machine learning with multi-source remote-sensing data (including Sentinel-2 and Landsat-8) has become the mainstream technical approach for retrieving key water-quality parameters like chlorophyll-a and total suspended solids. In terms of impact, highly cited publications significantly steer the direction of disciplinary development through methodological innovation or theoretical breakthroughs. Research focuses on the practical monitoring needs of inland and coastal waters, reflecting a clear application-oriented approach. Future trends indicate that scholars continue to address core challenges, such as algorithm generalizability and model transferability, while actively promoting interdisciplinary integration of remote-sensing technology with fields such as water environmental science and ecology to meet the urgent demands of complex water-system governance.

In the article, Sagan analyzes the latest advancements in water-quality remote sensing, discusses the limitations of existing systems and estimation methods, and proposes future improvement suggestions. The research findings indicate that proximal and satellite sensors hold significant potential for accurately estimating optically active parameters, while remote sensing of non-optically active parameters still faces challenges [30]. Pahlevan proposed the Mixture Density Network machine learning model, relying on Sentinel-2 and Sentinel-3 satellite data, and successfully achieved the seamless retrieval of chlorophyll-a concentration in inland and coastal waters [31].

### 3.6. Analysis of Keywords Distribution of Published Articles

**Figure 4:**
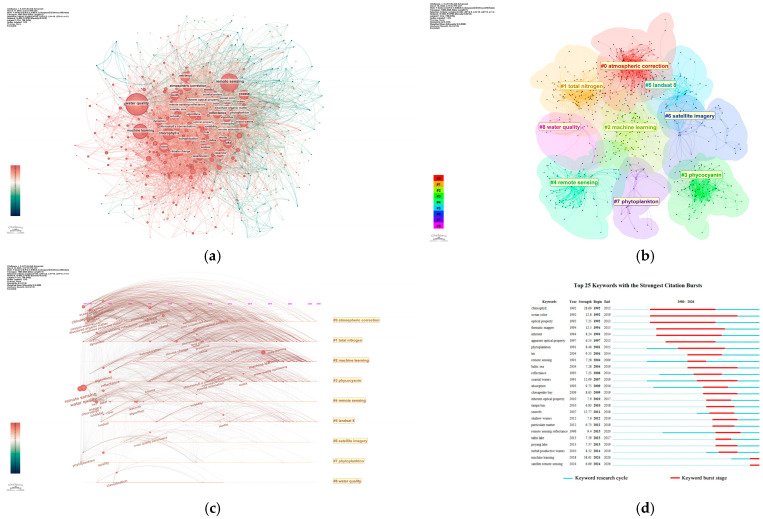
Analysis of the keyword distribution of global publications in the field of remote-sensing inversion of water-quality parameters: (**a**) keyword co-occurrence map; (**b**) keyword clustering map; (**c**) keyword timeline map; (**d**) keyword burst map.

Keyword analysis is an important method for identifying research hotspots in a field. With the help of CiteSpace, this study constructed a keyword co-occurrence visualization map for the field of remote-sensing inversion of water-quality parameters from 1980 to 2026, using a 1-year time slice (see Figure 4a). Table 3 presents the top 15 high-frequency keywords in this field. Based on keyword frequency analysis, the core hot keywords in this field include: water quality, remote sensing, algorithms, chlorophyll-a, model, coastal, atmospheric correction, machine learning, and reflectance. The above analysis clearly delineates the research trajectory in the field of remote-sensing inversion for water-quality parameters. This field employs remote-sensing technology as its core methodology and has established a comprehensive research paradigm characterized by “physical mechanisms as the foundation, data-driven innovation, and addressing core issues in typical scenarios.” Specifically, the research advances along two main lines: First, in terms of technical methods, it encompasses the entire chain from atmospheric correction and reflectance calculation based on physical mechanisms to the construction of data-driven inversion models represented by machine learning, reflecting an evolutionary path of inheritance and integration between traditional physical methods and modern artificial intelligence technologies. Second, in terms of research focus, it highly concentrates on key water-quality parameters, with chlorophyll-a as a representative example, and uses coastal waters as a typical application scenario, highlighting the close integration of research with practical needs. Overall, this field has developed a systematic research framework that spans from theoretical methods to practical applications.

To further clarify the thematic structure and evolutionary context of the field, this study conducted an in-depth investigation by combining the keyword clustering analysis results in Figure 4b and the clustering labels in Table 4, supplemented by the cluster timeline map in Figure 4c. The network is clustered into nine research themes in total, with the corresponding clustering labels listed in Table 4. The cluster numbers range from #0 to #8, where a smaller number indicates a larger quantity of keywords contained in the cluster. Through keyword cluster analysis, it has been found that the research topics in this field can be summarized into three core clusters: the first cluster focuses on technical methods, covering key aspects such as atmospheric correction and machine learning; the second cluster centers on core monitoring targets and parameters, including total nitrogen, phycocyanin, phytoplankton, and water quality; the third cluster corresponds to data sources and platforms, represented by remote-sensing, Landsat 8, and satellite imagery. This structure clearly reflects a “methods–objects–data” tripartite research framework. From the perspective of connection relationships, most keyword connections are concentrated within clusters, while clusters such as #0, #5, #2, and #1 have numerous cross-cluster connections. This indicates that these research directions are highly similar in terms of thematic scope, with high co-citation frequencies in the field, thus showing strong correlation in the field of remote-sensing inversion of water-quality parameters. In the cluster timeline map, the color of each growth ring corresponds to the citation time interval, and the thickness of the growth ring is positively correlated with the citation frequency in the corresponding period. Further analysis based on this characteristic reveals that cluster #0 represents the core research direction of the field, covering atmospheric correction and its sub-directions, such as visible infrared-imaging radiometer suite and MODIS. It has been continuously active since the initial stage of research, confirming that atmospheric correction technology is the core supporting foundation of this field.

The keyword burst detection map (see Figure 4d) reveals the burst dynamics of 25 keywords from 1980 to 2026. The concentrated emergence of such keywords usually corresponds to a period of rising attention in the field, driven by changes in research trends or technological breakthroughs. Among them, the burst strengths of “machine learning”, “chlorophyll”, “ocean color”, “seaWiFS”, “thematic mapper”, and “coastal waters” all exceed 10, indicating that these directions have experienced explosive growth in attention. In particular, the burst duration of “chlorophyll”, “ocean color”, “optical property”, “thematic mapper”, and “inherent” are as long as 20 years, entering a period of explosive research starting from 1992. This result indicates that remote-sensing-inversion research centered on optical properties, such as chlorophyll and ocean color, has consistently held a core theoretical position in this field since its emergence in the early 1990s, and has gradually evolved into a long-term research focus with sustained and foundational significance. Since 2013, research focusing on typical inland water bodies, such as Taihu Lake and Poyang Lake, has begun to emerge intensively. Compared to the coastal-water research that started in 1991, this trend appeared relatively late. This phenomenon clearly indicates that the scope of water-quality remote-sensing-inversion research is systematically expanding from coastal areas to inland waters, fully reflecting the continuous extension of the field’s application space and the ongoing deepening of practical scenarios. Among the burst keywords in the past two years, terms such as “machine learning” and “satellite remote sensing” have shown significant emergence. Notably, “machine learning” has reached a burst strength of 38.61, ranking first among all keywords in burst intensity. This indicates that machine learning has become the most influential cutting-edge hotspot in the current field of remote-sensing inversion of water-quality parameters.

## 4. Discussion

This study conducted a bibliometric analysis of existing research in the field of remote-sensing inversion of water-quality parameters by CiteSpace, focusing on an in-depth analysis of the following key issues: Which countries and research institutions have ranked among the top in research activity in this field? What are the most influential journals in the field? What constitutes the classic research outputs of remote-sensing inversion of water-quality parameters? What characteristics are presented in the mainstream research topics and development trends of this field over the past forty-five years?

Given the long cycle of data collection and collation, as well as the inevitable time lag in the academic dissemination of new research findings, bibliometric analysis can effectively identify the long-term research hotspots and evolutionary trends in this field, yet it is unable to cover innovative emerging research directions in a real-time and comprehensive manner. Based on this, combined with the results of keyword co-occurrence and the clustering and burst detection derived from CiteSpace analysis, this study systematically reviewed the relevant research papers recently published in this field, and the core evolutionary directions of the remote-sensing inversion of water-quality-parameters field in recent years can be summarized into three dimensions: the transition of data sources, the iteration of inversion models, and the shift of typical research areas.

### 4.1. Transition of Remote-Sensing Data Sources

As the most widely used remote-sensing data in monitoring applications, multispectral images have been employed by scholars at home and abroad to conduct extensive research on water-quality-parameter retrieval. Common multispectral remote-sensing data for water-quality monitoring include Landsat series satellite data, Sentinel satellite data, as well as domestic Gaofen (GF) series satellite data and Ziyuan-1 02C satellite data [32,33]. Due to resolution limitations, multispectral remote-sensing water-quality retrieval models are mainly constructed using empirical methods, making them more suitable for specific periods or water bodies. Hyperspectral satellite remote-sensing data are acquired by hyperspectral sensors mounted on satellites, featuring high resolution and large data volume. They can capture continuous surface-feature spectral information and provide more refined remote-sensing data. Typical hyperspectral data include Hyperion data from the United States (the world’s first satellite-borne hyperspectral sensor), Compact Airborne Spectrographic Imager/Shortwave infrared Airborne Spectrographic Imager (CASI/SASI) sensors from Canada, and HyperSpectral Imager (HSI) data from China’s HJ-1A satellite, all of which have been applied in water-quality retrieval research [34]. With high-spectral resolution and abundant data bands, hyperspectral data overcome the shortcomings of previous remote-sensing data in spectral resolution, enabling the accurate and optimal selection of water-quality retrieval methods and distinguishing spectral mixing differences in multispectral data. This significantly improves the accuracy of water-quality-parameter retrieval and demonstrates excellent application potential [35].

In recent years, high-spatial-resolution remote-sensing data, such as various airborne remote-sensing data and ground-measured spectral data, have been widely used. With the rapid development of UAV technology, lightweight UAV systems equipped with multispectral cameras, hyperspectrometers, infrared sensors, and lidar have emerged. High-resolution data acquired by these airborne systems show significant improvements in imaging timeliness and quality, gradually becoming an important data source for remote-sensing monitoring of water quality in small water bodies [36]. Typical UAV-borne sensors include Airborne Visible InfraRed Imaging Spectrometer (AVIRIS) from the United States, CASI from Canada, and the Pushbroom Hyperspectral Imager (PHI) from China. In addition to airborne spectrometers, portable spectrometers have also played a certain role in water-quality monitoring due to their convenience and flexibility. In general, non-satellite spectral remote-sensing data offer advantages of higher spectral and spatial resolution, and can continuously and accurately depict the spectral curves of surface features. Compared with satellite data, non-satellite remote-sensing data are less affected by atmospheric interference but have higher data-acquisition costs and smaller coverage, resulting in relatively insufficient comprehensive observation capabilities.

Each type of remote-sensing data has more or less limitations in depicting or expressing surface feature characteristics, and it is difficult for a single data source to meet the needs of water-quality remote-sensing monitoring in most study areas. Therefore, some scholars have recently begun to attempt fusing remote-sensing data from different sources for water-quality monitoring [37]. Data fusion can yield more accurate and abundant information than any single data source, thereby providing more reliable basic data for water-quality-parameter retrieval and monitoring. Feng [38] used GF-1 WFV data (with a short revisit cycle and high spatial resolution) and Landsat 8 OLI data (with high spectral resolution) to explore the optimal combination method for collaborative retrieval of WFV and OLI data, aiming to improve retrieval accuracy. Dona [39], Essam [40], and other scholars have attempted to obtain water-quality parameters by integrating satellite remote-sensing data fusion and artificial intelligence methods, using this approach to address several issues in inland water-quality retrieval. The information pertaining to different types of remote-sensing data is compared in Table 5.

### 4.2. Iteration of Remote-Sensing Retrieval Methods

Traditional water-quality retrieval methods are roughly classified into three categories: analytical models, empirical models, and semi-empirical models. Among these, analytical models are constructed based on physical mechanisms, such as radiative transfer theory, offering clear physical significance. They systematically reveal the intrinsic response mechanisms between water-quality parameters and spectral signals, providing a theoretical framework for understanding the optical properties of water bodies. However, in practical applications, such models are often limited by factors such as the complexity of water constituents and the idealization of boundary conditions. Empirical models rely on the empirical relationships between water-quality parameters measured synchronously in situ and remote-sensing reflectance, offering relatively simple operation and the ability to quickly obtain results when data are sufficient and water bodies are relatively stable. Nevertheless, their universality is low, and differences in water-body characteristics across different regions may easily render the models ineffective. Although semi-empirical models integrate certain advantages of both analytical and empirical models—considering physical mechanisms to a certain extent while optimizing model parameters using measured data—they still suffer from drawbacks such as complex model construction, high requirements for data volume and quality, and difficulty in accurately reflecting the complex and variable characteristics of water quality. Overall, traditional methods hold significant value in terms of mechanistic interpretability. However, when dealing with high-dimensional, nonlinear, and interference-prone real-world remote-sensing data, they still face notable challenges in terms of accuracy and adaptability [41,42]. Yuan [43] established regression models between chlorophyll-a, total phosphorus (TP), and total nitrogen (TN) using Landsat 8 data, and the final results indicated that the binomial algorithm performed optimally. Xu [44] constructed linear regression models to retrieve the concentrations of inorganic nitrogen and dissolved inorganic phosphorus using Beijing-1 multispectral images and data from 60 sampling points near the Sheyang Estuary. The results showed that the cubic polynomial model achieved high retrieval accuracy.

In recent years, with the rise in data-driven approaches, artificial intelligence algorithms have gradually become a research hotspot in water-quality remote-sensing inversion. The physical mechanisms and spectral knowledge established by traditional models provide interpretable structural priors and feature constraints for machine learning models, such as input features, band combinations, and physical regularization terms constructed based on bio-optical principles. This enhances the models’ generalization ability and mechanistic consistency in complex environments. Compared with the aforementioned traditional empirical algorithms, AI algorithms possess powerful nonlinear fitting capabilities and robustness, enabling them to effectively cope with complex and diverse background effects from water surfaces, combinations of water-quality parameters and sediments, and explore implicit relationships to improve retrieval accuracy. Currently, algorithms such as Convolutional Neural Networks (CNNs) [45,46], Support Vector Regression (SVR) [47,48], and random forests (RFs) [49,50] have been widely applied in water-quality-parameter inversion. They effectively capture the nonlinear relationships between water-quality parameters and remote-sensing signals while suppressing background noise interference. In addition, Extreme Gradient Boosting (XGBoost) regression and Categorical Boosting (CatBoost) regression have also been increasingly applied in water-quality retrieval. XGBoost integrates regularization techniques to control model complexity and exhibits considerable capabilities in handling missing values and feature engineering. CatBoost is specifically designed to process categorical features and automatically performs feature transformation without the need for additional preprocessing steps, which gives it advantages in certain fields [51,52]. These AI algorithms not only inherit the optical mechanistic insights from traditional physical models but also break through the modeling bottlenecks of conventional methods in complex scenarios through data-driven approaches. This drives the development of water-quality remote sensing toward high precision, intelligence, and the integration of mechanisms and data. A comparison of different remote-sensing-inversion methods is presented in Table 6. Pang [53] established four mathematical statistical models and an XGBoost model to retrieve water ammonia nitrogen concentrations using three phases of UAV multispectral image data. Comparative experiments showed that the XGBoost model outperformed the mathematical statistical models, demonstrating strong fitting ability and high prediction accuracy, with the retrieval accuracy (R^2^) of ammonia nitrogen in all three phases exceeding 0.84. Wang [54] proposed a remote-sensing retrieval model for nitrate and phosphate concentrations using backpropagation neural networks (BPNNs) in the coastal waters of the East China Sea. The retrieval accuracies (R^2^) of nitrate and phosphate concentrations were 0.98 and 0.83, respectively. Yu [55] developed a stacked random forest (SRF) model for surface seawater nitrate (SSN) using MODIS data as the data source and the central and southern regions of the California Current System as the study area to simulate the relationship between SSN and relevant environmental parameters. The retrieval accuracy (R^2^) of this model was 0.87. The comparison of different remote-sensing-inversion methods in Table 6.

### 4.3. Shift of Typical Research Areas

Based on the burst detection results of keywords and systematic combing of the relevant literature, it can be found that in 2004, the typical research areas in this field focused on coastal waters, with the research core centered on the exploration of water color characteristics in coastal waters. The research scope mainly covered typical coastal bay waters—including Shenzhen Bay, the Pearl River Estuary, Hangzhou Bay, Chesapeake Bay in the United States, the coastal waters of the Baltic Sea, and Moreton Bay in Australia—whereas inland waters have exhibited a prominent burst characteristic since 2013, which indicates a sharp surge in research achievements related to inland waters. This shift in the focus of research areas is the combined effect of policy guidance, technological breakthroughs, practical demands, and disciplinary development. Specifically, the national-level policies that continuously strengthen the protection of inland aquatic ecology have provided directional guidance for the research on inland water bodies. Technological innovations in hyperspectral remote sensing, UAV monitoring, and machine learning algorithms have broken through the technical bottlenecks caused by the complex optical characteristics and high inversion difficulty of inland water bodies. The increasingly prominent ecological problems, such as eutrophication and nitrogen and phosphorus pollution in inland rivers, lakes, and reservoirs, have spawned the demand for large-scale and high-precision monitoring research. Meanwhile, a mature theoretical and methodological system has been formed in the research on water-quality monitoring of coastal waters, which has prompted scholars to gradually turn their attention to inland water bodies as an important research gap and hotspot in this field.

Reflecting this shift in research focus, relevant research practices in both coastal and inland waters are illustrated through typical case studies outlined below: Zhao [65] conducted transparency inversion in research areas, including Lake Chagan. They monitored the spatiotemporal pattern changes in water transparency in Lake Chagan over a long time scale via remote sensing, and further analyzed the variation characteristics and regularities of water transparency. Chen [66] used Sentinel-2 satellite images to invert the water-quality indicators of Lake Gaoyou, and obtained the spatiotemporal distribution characteristics of chlorophyll-a (Chl-a) and total suspended solids in the lake. Taking Taihu Lake and its surrounding rivers as the research area, Lin [67] constructed a remote-sensing-inversion model for permanganate index (COD_Mn_) concentrations in Taihu Lake and adjacent rivers based on Sentinel-2 multispectral images and synchronous in situ COD_Mn_ data by adopting multiple linear regression and machine learning methods. This study demonstrated the feasibility of remote-sensing inversion for COD_Mn_, a non-optically active water-quality parameter. Based on MODIS satellite data and 4038 sets of in situ measured data, Zhu [68] inverted the water quality of coastal waters in the northern South China Sea using machine learning methods such as Classification and Regression Tree and XGBoost, and the results indicated that the XGBoost algorithm yielded the optimal performance.

## 5. Conclusions

### 5.1. Summary of Knowledge-Graph Analysis Results Based on CiteSpace

As revealed by the bibliometric analysis via CiteSpace, research on the remote-sensing inversion of water-quality parameters has exhibited distinct interdisciplinary characteristics over the past forty-five years, yielding fruitful outcomes that integrate academic research with practical applications. The annual publication output of this field has gone through three prominent stages: the initial exploration stage (1980–2000), the slow growth stage (2001–2015), and the rapid explosion stage (2016–2026). The annual number of publications has exceeded 100 since 2018 and reached 569 in 2025. This vigorous development of the field can be attributed to technological breakthroughs, policy-driven impetus, and interdisciplinary integration. In light of the current trend, the field is set to embrace an even more thriving development.

In the global ranking of publications by country, China ranks first, followed by the United States and India, with research coverage extending across the globe. Collaborative networks have been formed at both national and institutional levels. The Chinese Academy of Sciences, Wuhan University, and the NASA serve as the core publishing institutions, presenting a research pattern featured by the leadership of core organizations and multi-agent collaboration. The field is characterized by distinctive core journals and the classic literature. Highly cited papers focus on chlorophyll-a inversion and the application of Sentinel satellite data, and machine learning algorithms have become the mainstream technical methods, laying a solid foundation for research in this field.

Analyses of keyword co-occurrence, clustering, and burst detection show that research hotspots concentrate on water quality, remote sensing, chlorophyll-a, atmospheric correction, and machine learning, forming two main research threads: the optimization of preprocessing technologies and the monitoring of water-quality parameters. Research scenarios have expanded from coastal waters to inland water bodies, and algorithm optimization has emerged as an emerging research hotspot in recent years. Overall, the application scope and technical system of this field are continuously developing and improving.

### 5.2. Research Challenges and Prospects

Remote-sensing inversion of water-quality parameters is a vital research direction at the interdisciplinary frontier of remote-sensing technology, environmental science, and water resources management, as well as a key technical support for the precise monitoring of water resources and ecological environment governance. This study is a bibliometric-based narrative review that systematically sorts out the research achievements in the global field of remote-sensing inversion of water-quality parameters over the past forty-five years, aiming to present the research status, development trends, and core hotspots of this field for researchers, and provide references for subsequent academic exploration and practical applications. Multi-source data fusion and artificial-intelligence-driven optimization of inversion models constitute the core research hotspots in this field, spawning numerous innovative interdisciplinary designs and studies integrating remote-sensing science, computer technology, and environmental science. It is found that the advancement of multi-source remote-sensing data-fusion technologies (including satellite, UAV, and ground-measured spectral data) has provided richer data sources and more reliable basic information for the remote sensing inversion of water quality parameters. Intelligent algorithms represented by machine learning and deep learning have broken through the limitations of traditional inversion models, significantly improved the inversion accuracy of water quality parameters, and offered promising solutions for achieving large-scale and high-precision dynamic monitoring of water resources. However, the field is currently confronted with three core challenges: first, the adaptability of multi-source remote-sensing data-fusion technologies is insufficient, and existing methods are difficult to meet the practical demands of water-quality monitoring; second, the optimization of water-quality-inversion models is not in-depth enough, with inadequate integration of geospatial and thematic factors, leading to the need for further improvement in the universality and adaptability of the models; third, the research on inland water bodies lacks a systematic approach, and there is a shortage of inversion models adapted to their complex optical characteristics. In response to the above research gaps, future research in this field can be advanced with a focus on the following three aspects:

(1) Conduct in-depth research on remote-sensing data-fusion methods. With the continuous advancement of remote-sensing technology, an increasing number of remote-sensing images have become available for water-quality monitoring. However, the sensors used to acquire these images differ in design objectives and observation characteristics: satellite data feature a wide coverage but suffer from limited spatiotemporal resolution and high susceptibility to weather conditions; UAV images boast high resolution and flexible acquisition, being free from weather interferences such as cloud cover, yet are constrained by a narrow observation range, poor endurance, and high costs. In this regard, different datasets exhibit respective strengths and weaknesses in characterizing water-quality features. Therefore, data fusion has become a key approach to improving the efficiency of water-quality monitoring. Existing fusion methods include the Curvelet transform, wavelet transform, and weighting-based methods, but their practical application in the field of water-quality monitoring remains inadequate. Future research needs to conduct in-depth exploration of image data-fusion methods tailored to the demands of water-quality monitoring. By fusing image data captured by different sensors—such as the integration of satellite-borne, airborne remote-sensing data and ground-measured spectral data, as well as remote-sensing data with high spatial resolution and high spectral resolution—we can fully exploit the unique advantages of various sensors, construct an air–space–ground collaborative fusion framework, and develop algorithms that balance spectral matching and spatial detail preservation. Differentiated fusion strategies should be designed to drive the evolution of multi-source data from simple superposition to deep coupling, which can better characterize water-quality features and provide more reliable fundamental data for water-quality remote-sensing inversion.

(2) Further optimize water-quality-inversion models. Previous research on water-quality inversion has over-relied on the spectral characteristics of water quality, while neglecting the auxiliary effects of geospatial factors (e.g., distance to lake shorelines, distance to river estuaries or lake inlets) and thematic factors (e.g., water temperature, flow velocity, and water depth) on water-quality inversion. Qi [69] incorporated geospatial elements into his research and developed the GTNNWR model, which can accurately capture the spatiotemporal dynamic changes in dissolved silicate (DSi) in coastal seas, thereby providing remote-sensing early warning signals for the occurrence of coastal red tides. Future research can integrate the aforementioned geospatial and thematic factors to further improve the inversion accuracy of water-quality remote sensing. On the other hand, given the great potential of machine learning methods in enhancing the inversion accuracy of water-quality parameters, it is recommended to further strengthen research on machine-learning-based water-quality-inversion methods. In the future, existing research data can be utilized to train and construct large artificial intelligence models for the field of water-quality inversion, so as to improve the universality and adaptability of water-quality-inversion models.

(3) Strengthen research on inland water bodies. Research on coastal water bodies started early and has formed a well-established technical system, while studies on inland water bodies have boomed in recent years with a growing number of relevant achievements, yet the systematic nature of the research remains to be improved. Future research should focus on targeted investigations of inland water bodies, develop specific water-quality-inversion models in combination with their unique water body characteristics, and address the technical challenges in remote-sensing monitoring of inland water quality. In the meantime, efforts should be made to advance the cross-integration of research on coastal and inland water bodies, explore the common rules and individual differences in inversion in the two water types, and realize mutual learning and integration of technical methods. This will help build a land–sea coordinated water-quality remote-sensing monitoring system, and further provide scientific support for the comprehensive management of the water ecological environment and regional sustainable development.

## Figures and Tables

**Table 1:** Top 10 co-cited journals of the studied publications from 1980 to 2026.

Rank	Number of Articles Published	Year	Journal
1	2267	1983	*Remote Sensing of Environment*
2	1841	1987	*International Journal of Remote Sensing*
3	1775	2011	*Remote Sensing*
4	1620	1989	*Science of the Total Environment*
5	1193	1989	*Limnology and Oceanography*
6	1023	1991	*IEEE Transactions on Geoscience and Remote Sensing*
7	1005	2014	*Water* (Switzerland)
8	997	1989	*Water Research*
9	996	1986	*Applied Optics*
10	897	1987	*Journal of Geophysical Research: Oceans*

**Table 2:** Top 10 co-cited studies of the publications from 1980 to 2026.

Rank	Freq	Year	Title	Citations
1	216	2020	Monitoring Inland Water Quality Using Remote Sensing: Potential and Limitations of Spectral Indices, Bio-optical Simulations, Machine Learning, and Cloud Computing	481
2	186	2020	Seamless Retrievals of Chlorophyll-a from Sentinel-2 (MSI) and Sentinel-3 (OLCI) in Inland and Coastal Waters: A Machine-Learning Approach	436
3	153	2020	A Machine Learning Approach to Estimate Chlorophyll-a from Landsat-8 Measurements in Inland Lakes	327
4	135	2022	A Review of Remote Sensing for Water Quality Retrieval: Progress and Challenges	268
5	110	2020	Research Trends in the Use of Remote Sensing for Inland Water Quality Science: Moving Towards Multidisciplinary Applications	228
6	101	2020	Robust Algorithm for Estimating Total Suspended Solids (TSS) in Inland and Nearshore Coastal Waters	207
7	100	2021	ACIX-Aqua: A Global Assessment of Atmospheric Correction Methods for Landsat-8 and Sentinel-2 over Lakes, Rivers, and Coastal Waters	274
8	96	2022	Simultaneous Retrieval of Selected Optical Water Quality Indicators from Landsat-8, Sentinel-2, and Sentinel-3	182
9	93	2016	A Comprehensive Review on Water Quality Parameters Estimation Using Remote Sensing Techniques	766
10	86	2021	Quantification of Chlorophyll-a in Typical Lakes across China using Sentinel-2 MSI Imagery with Machine Learning Algorithm	145

**Table 3:** Top 15 keywords by frequency for the studied publications from 1980 to 2026.

Rank	Count	Year	Keywords
1	1015	1991	water quality
2	910	1991	remote sensing
3	419	1991	chlorophyll a
4	365	1992	model
5	318	1993	coastal
6	311	2001	retrieval
7	289	2001	atmospheric correction
8	285	2018	machine learning
9	283	1995	reflectance
10	265	1994	lake
11	237	1994	quality
12	232	1994	algorithms
13	189	1991	algorithm
14	189	1991	phytoplankton
15	188	2003	inland

**Table 4:** Classification of keyword clusters for the studied publications from 1980 to 2026.

Cluster ID	Name	Size	Silhouette	Top Terms
0	atmospheric correction	160	0.539	water quality; Yangtze River; Lake Taihu; visible infrared imaging radiometer suite; MODIS|atmospheric correction; gold mining; temporal analysis; Brazilian Amazon; turbid rivers
1	total nitrogen	151	0.509	water quality; air pollutants; spatial influence analysis; graph convolution network; artificial neural networks models|machine learning; non-point-source pollution; tropical drinking water source; multimodal model; meteorological lag effects
2	machine learning	115	0.594	machine learning; random forest; biological system modeling; spatial resolution; Secchi disk depth|water quality; deep learning; water pollution; water resources; environmental monitoring
3	phycocyanin	102	0.579	water quality; water clarity; ocean color satellite images; coastal environment; remote-sensing modeling|machine learning; Yangtze River Basin; temporal representativeness; sediment plume; Lake Pontchartrain
4	remote sensing	90	0.688	water quality; neural network; Chagan Lake; algal growth; algal biofuel|machine learning; coastal waters; biological system modeling; data models; predictive models
5	Landsat 8	57	0.616	water quality; estuary health; suspended sediment concentration; semianalytical algorithms; brackish lake|machine learning; transfer learning; semi-supervised regression; Sidi Moussa Lagoon; Atlantic Moroccan Coast
6	satellite imagery	45	0.852	water-quality parameters; thematic mapper; bio-optical property; environmental monitoring; total phosphorus|water quality; calibration; turbidity; chlorophyll a; total suspended matter
7	phycocyanin	102	0.579	water quality; water clarity; ocean color satellite images; coastal environment; remote-sensing modeling|machine learning; Yangtze River Basin; temporal representativeness; sediment plume; Lake Pontchartrain
8	remote sensing	90	0.688	water quality; neural network; Chagan Lake; algal growth; algal biofuel|machine learning; coastal waters; biological system modeling; data models; predictive models

**Table 5:** Comparison of information from different remote-sensing data.

Data Type	Usage Scale	Advantages & Disadvantages
Multispectral	Medium scale, covering tens to hundreds of square kilometers; suitable for regional monitoring of large water bodies (e.g., lakes, oceans).	**Advantages:** Easy sensor acquisition, low cost; multi-band data supports model construction for macro analysis. **Disadvantages:** Low spectral resolution, long revisit period; hard to monitor dynamic changes in a timely manner.
Hyperspectral	Small-to-medium scale, usually covering several hundred to several thousand square kilometers; applicable to small lakes, estuaries, etc.	**Advantages:** High spectral resolution, enabling accurate detection of water-quality parameters and identification of trace substances. **Disadvantages:** Large data volume (hard to process), high cost; limited spatial resolution; complex interpretation.
UAV	Small scale, generally covering several square meters to several thousand square kilometers; used for small water bodies (e.g., small ponds, urban rivers).	**Advantages:** High flexibility; real-time high-resolution data acquisition suitable for emergency monitoring. **Disadvantages:** Limited flight range/duration; weather-dependent; complex data processing.
Multi-source Data	Medium-to-large scale, covering hundreds to tens of thousands of square kilometers; applicable to large river basins, broad oceans, etc., for comprehensive monitoring.	**Advantages:** Integrates multi-data advantages; monitoring is more comprehensive/accurate with reliable results. **Disadvantages:** Complex fusion technology; difficult management/processing; errors/uncertainties affect analysis.

**Table 6:** Comparison of different remote-sensing-inversion methods.

Remote-Sensing-Inversion Methods		Applicable Scenario	Sample Requirement	Accuracy (R^2^)	Computational Cost	Advantages & Disadvantages
Traditional Inversion Methods	Analytical Model [56]	Large-scale water bodies (e.g., lakes, oceans) for long-term monitoring	100–300	0.70–0.85	High	**Advantages:** Strong theoretical foundation, wide applicability, high interpretability; **Disadvantages:** Complex model, difficult parameter acquisition, requires large amounts of measured data
	Empirical Model [57]	Small-scale areas for short-term monitoring (e.g., small rivers)	20–50	0.60–0.8	Low	**Advantages:** Simple construction, low computational load, high accuracy under specific conditions; **Disadvantages:** Regional limitations, data-dependent, poor applicability when the environment changes
	Semi-Empirical Model [58]	Medium-scale areas for seasonal monitoring	50–200	0.70–0.85	Medium	**Advantages:** Combines the advantages of both models, simultaneous improvement in accuracy and applicability; **Disadvantages:** Regional adaptability issues, requires certain data quality and quantity
AI Algorithms	Convolutional Neural Network (CNN) [59]	High-resolution images, complex nonlinear water-quality parameters	>500	0.85–0.95	High	**Advantages:** Strong feature extraction capability, suitable for complex data patterns; **Disadvantages:** High computational resource requirements, poor model interpretability
	Support Vector Regression (SVR) [60]	Small-to-medium scale, nonlinear, non-normal water-quality-parameter inversion	50–300	0.80–0.9	Medium–High	**Advantages:** Good performance with small samples, strong resistance to noise and outliers; **Disadvantages:** Complex computation, cumbersome parameter adjustment, poor interpretability
	Random Forest Regression (RF) [61]	Multi-parameter, high-dimensional scenarios	100–500	0.80–0.9	Medium	**Advantages**: Good generalization, not prone to overfitting, can handle outliers and missing values; **Disadvantages:** Limited interpretability
	Extreme Gradient Boosting (XGBoost) [62]	Large-scale, high-complexity water-quality-parameter inversion	100–500	0.85–0.95	Medium–High	**Advantages:** Optimal timeliness, anti-overfitting, strong feature-processing capability; **Disadvantages:** Slow training speed, many parameters, complex parameter tuning, poor interpretability
	Categorical Boosting (CatBoost) [63]	Water-quality data containing categorical features	100–400	0.85–0.92	Medium	**Advantages:** Handles categorical features, more accurate prediction, fast training; **Disadvantages:** Poor interpretability, hyperparameters affect performance
	Backpropagation Neural Network (BPNN) [64]	Complex nonlinear, non-normal water-quality-parameter inversion	>500	0.80–0.95	High	**Advantages:** Good nonlinear fitting, can learn complex functions; **Disadvantages:** Slow convergence to local optimum, parameter-sensitive

## Data Availability

No new data were created or analyzed in this study.

## Abbreviations

The following abbreviations are used in this manuscript:

UAV	Unmanned Aerial Vehicle
WOSCC	Web of Science Core Collection
LSI	Latent Semantic Indexing
NASA	National Aeronautics and Space Administration
CASI	Compact Airborne Spectrographic Imager
XGBoost	Extreme Gradient Boosting
CatBoost	Categorical Boosting
SSN	Surface Seawater Nitrate
COD_Mn_	Permanganate Index
